# Individual Preventive Behaviors of COVID-19 and Associated Psychological Factors Among Chinese Older Adults: A Cross-Sectional Online Survey

**DOI:** 10.3389/fpsyg.2022.827152

**Published:** 2022-03-21

**Authors:** Yanping Duan, Chun Hu, Zhihua Lin, Wei Liang, Borui Shang, Julien Steven Baker, Jiali He, Yanping Wang

**Affiliations:** ^1^Department of Sport, Physical Education and Health, Hong Kong Baptist University, Kowloon, Hong Kong SAR, China; ^2^Center for Health and Exercise Science Research, Hong Kong Baptist University, Kowloon, Hong Kong SAR, China; ^3^College of Health Science, Wuhan Institute of Physical Education, Wuhan, China; ^4^Student Mental Health Education Center, Northwestern Polytechnical University, Xi’an, China; ^5^Sport Section, Wuhan University, Wuhan, China; ^6^Department of Kinesiology, Hebei Institute of Physical Education, Shijiazhuang, China

**Keywords:** COVID-19 pandemic, preventive behaviors, demographic factors, psychological factors, older adults

## Abstract

**Purpose:**

Older adults aged 60 years and above are classified as being of high-risk for infection during the COVID-19 pandemic. This study aimed to investigate the associations of psychological factors (motivational factors: risk perception, health knowledge, attitude, subjective norm, motivational self-efficacy, and intention; volitional factors: volitional self-efficacy, planning, and action control) of preventive behaviors with three preventive behaviors (hand washing, facemask wearing, and social distancing) among Chinese older adults during the COVID-19 pandemic.

**Methods:**

A cross-sectional questionnaire survey was administered *via* SOJUMP, a widely used online survey platform in China. A total of 928 older adults (mean = 67.24 years, age range: 60–90 years, SD = 6.43, 55.9% females) were recruited using a snowball sampling approach from Hubei Province (*n* = 667) and outside Hubei Province (*n* = 261) in China during May 18, 2020 to June 7, 2020. Multiple hierarchical regressions were conducted with four models to examine the association between demographic, past behavior, psychological factors and each preventive behavior.

**Principal Findings:**

All three preventive behaviors in older adults increased dramatically during the pandemic of COVID-19. Gender, living status, educational level, past behavior, health knowledge, intention and planning significantly predicted hand washing behavior, *R*^2^ = 0.395, *F*(10, 927) = 54.372, *p* < 0.001. Gender, education level, important others (e.g., family members or friends) infection, past behavior, health knowledge, planning and action control significantly predicted mask wearing behavior, *R*^2^ = 0.202, *F*(10, 927) = 23.197, *p* < 0.001. Living place, past behavior and health knowledge significantly predicted social distancing behavior, *R*^2^ = 0.204, *F*(9, 927) = 26.201, *p* < 0.001.

**Major Conclusions:**

Past behavior and health knowledge predicted all three preventive behaviors. Planning was an important psychological factor for both hand washing and mask wearing behaviors. All those critical demographic and psychological factors are critical for future interventions to facilitate older adults to comply with three preventive behaviors in daily life and to stay healthy during the COVID-19 pandemic.

## Introduction

Since its initial detection in late December 2019, the coronavirus disease (COVID-19) pandemic has been the most severe public health emergency, which has caused over 260 million confirmed cases and more than 5.19 million deaths worldwide inclusive of 28 November 2021 [Center for Systems Science and Engineering (CSSE), 2021]. Older adults aged 60 years and above are classified as the high-risk group during the pandemic as they are more susceptible and vulnerable to infectious diseases due to decreased immune function and multi-morbidity (Kluge, 2020; Song et al., 2020). The risk of infection progressing to severe cases after confirmation of COVID-19 increases with age in older adults (Chinese Center for Disease Control and Prevention, 2020). In addition, high hospitalization rates and high fatality rates among older adults are of great economic burden to the government.

Given that there is still not enough vaccination prevention for COVID-19 worldwide and in anticipation of rapidly mutating viruses which transitions may not be prevented by vaccinations, the everyday personal preventive actions, such as performing hand hygiene frequently, wearing facemasks and keeping secure social distance in public areas play a very important role in COVID-19 prevention for the general public (World Health Organization, 2020). Currently, older population’s preventive behaviors during COVID-19 are not fully understood globally. Little is known about how often and to what extent older people execute preventive actions. In addition, it is crucial to investigate the psychological factors associated with older adults’ preventive behaviors as these can provide intervention strategies for increasing behavioral compliance toward COVID-19 prevention in this population.

In order to maximize the prediction function of psychological factors towards behavior change, a comprehensive review of these factors is needed. In general, psychological factors of behaviors comprise motivational factors associated with behavior initiation and volitional factors associated with behavior maintenance. The Theory of Planned Behavior (TPB) identified specific motivational factors, including attitude (positive or negative evaluations towards the consequences of performing the intended behavior), subjective norm (perceived expectations of important others approving the intended behavior), perceived behavioral control (perception about being able to perform the intended behavior) and intention (Ajzen, 1985). These factors of TPB have shown significant predictions related to hand washing (Gaube et al., 2020; Zhang et al., 2020) and face mask wearing (Chung et al., 2018). In addition, previous research has also shown that health knowledge, as an important factor influencing the formation of behavior intention, was significantly associated with hand washing behavior among adults (Tao et al., 2013; Ajilore et al., 2017). Recent research has indicated that residents’ COVID-19 knowledge was significantly associated with their preventive behaviors including facemask wearing and keeping social distance (Zhong et al., 2020).

The Health Action Process Approach (HAPA) suggests attention to critical psychological factors both in the motivational phase and volitional phase (Schwarzer, 2008). During the motivational phase, risk perception (perceived susceptibility to a health threat in terms of both perceived vulnerability and perceived severity) and motivational self-efficacy (the beliefs about the ability to start the behavior even when facing difficulty) are considered important to form the intention of preventive behaviors (e.g., mask wearing; Zhou et al., 2016). After the intention is formed, self-regulatory strategies (e.g., planning, volitional self-efficacy and action control) need to be enacted to ensure an intention is realized, and once initiated, maintained in the volitional phase. In particular, planning includes action planning about “when,” “where,” and “how” to act as well as coping planning about how to overcome anticipated barriers to the action. Volitional self-efficacy contains beliefs about the capabilities to overcome barriers during the maintenance period and to regain control after a setback. Finally, action control comprises self-regulatory effort, self-monitoring, and awareness of behavioral standards to adjust behavior. The prediction function of planning, volitional self-efficacy and action control is supported in hand washing and facemask wearing research (Fernández et al., 2016; Zhou et al., 2016).

As the perceived behavior control (PBC) in TPB shares synonymous construct with motivational self-efficacy in HAPA (Schwarzer and Luszczynska, 2005), this study used motivational self-efficacy instead of PBC. After a review of the main psychological factors of behaviors, this study adopted the motivational factors including attitude, subjective norm, motivational self-efficacy, risk perception, health knowledge and intention, as well as the volitional factors including planning, volitional self-efficacy and action control. It is significant to adopt both motivational and volitional factors of preventive behaviors of COVID-19 in this study as most of the recent studies only focus on the motivational phase of preventive behaviors (Chen et al., 2020; Gaube et al., 2020; Zhong et al., 2020).

This study aimed to find the demographic characteristics of three preventive behaviors of COVID-19 (hand washing, facemask wearing, and social distancing) and to examine the associations of psychological factors with preventive behaviors among older adults during COVID-19 pandemic in China. Accordingly, it was hypothesized that the demographics, motivational factors (risk perception, health knowledge, attitude, subjective norm, motivational self-efficacy, and intention) and volitional factors (volitional self-efficacy, planning, and action control) of preventive behaviors would be positively associated with hand washing behavior (Hypothesis 1), with facemask wearing behavior (Hypothesis 2) and with social distancing behavior (Hypothesis 3), respectively. The present study may provide evidence of potentially modifiable variables for behavior change interventions aimed at promoting preventive behaviors among older adults during and beyond the COVID-19 pandemic.

## Materials and Methods

### Participants

Participants were recruited using a snowball sampling approach from Hubei Province of China (the most infected region during the COVID-19 pandemic in China) and outside Hubei Province of China. Data collection started on May 18, 2020 and was completed on June 7, 2020. We contacted 1,054 city community-dwelling older adults and finally recruited 928 valid participants (88% response rate) with 667 in Hubei Province and 261 outside Hubei Province. A total of 928 older adults aged from 60 to 90 years (mean = 67.24 years, SD = 6.43) met the eligibility criteria: (1) Aged 60 years and above; (2) Had access a mobile phone or laptop; (3) Had sufficient comprehension, reading, and listening skills in Chinese. For those participants who had difficulty in mobile phone operation, their family members or friends were invited to assist them to complete the survey.

### Procedures

The survey was constructed and administered using an online survey platform in China namely SOJUMP (Changsha Ranxing Information Technology Co., Ltd., China). All recruitment posters and the hyperlink for the survey were disseminated *via* a mobile Short Message Service (SMS) and WeChat (the most popular social media platforms in China). There were three approaches used for recruiting participants. (1) Relying on the researchers’ social networks, the eligible family members, friends and relatives of researchers were also invited. These initial participants then encouraged their friends to join the survey. (2) Researchers contacted the directors of community neighborhood committees in Wuhan and Xiaogan of Hubei Province, respectively, and sought their collaboration and support. Upon receiving the agreement of directors, researchers were permitted to enter into their community neighborhood WeChat groups to recruit eligible participants. (3) Researchers contacted officials who oversee the retirement in two universities in Wuhan of Hubei Province. With the support of officials, a recruitment poster and survey hyperlink were delivered to their internal WeChat group especially for retirement colleagues.

The duration of the online survey was around 15 min. Participants were asked to sign an informed consent form on the first page of the survey platform prior to completing the questionnaires. Ethical approval for the study was obtained from Hong Kong Baptist University Research Ethics Committee (REC/19-20/0490).

### Measurement

The demographic characteristics included age, gender, current living place, living status, marital status, education level, occupational status, and economic situation. Participants were also invited to report their self-evaluated health status, chronic disease situation, children, restriction/quarantine situation, COVID-19 infection situation of participants, COVID-19 infection situation of important others, body height (cm), and body weight (kg).

Three preventive behaviors before and during the COVID-19 pandemic (hand washing, mask wearing, and social distancing; Duan et al., 2021; Liang et al., 2021), motivational factors of preventive behaviors [risk perception (Perloff and Fetzer, 1986; Duan et al., 2017), health knowledge (Contzen and Mosler, 2015; Li et al., 2019), attitude (O’Boyle et al., 2001; Rosen et al., 2009), subjective norm (Ajzen, 2002; Chung et al., 2018), intention (Lippke et al., 2009; Duan et al., 2018; Liang et al., 2019), motivational self-efficacy (Zhou et al., 2016; Duan et al., 2018; Liang et al., 2019)] and volitional factors of preventive behaviors [volitional self-efficacy (Zhou et al., 2016; Duan et al., 2018; Liang et al., 2019), planning (Luszczynska and Schwarzer, 2003; Duan et al., 2018; Liang et al., 2019; Zhang et al., 2020), action control (Sniehotta et al., 2005; Zhang et al., 2020)] were measured by questionnaires. The details of the measurement tools are outlined in Table 1.

**Table 1:** Overview of the measurement tools regarding preventive behavioral and their motivational and volitional factors.

Name of variable	Origin	Detailed information
**Preventive behaviors** *Preventive behaviors during the COVID-19 pandemic*		
Hand Washing	Duan et al., 2021; Liang et al., 2021	Two items; 4-point Likert scale from (1) never to (4) always; “During the previous week, how frequently did you wash your hands with soap and water or alcohol-based hand rub (for at least 20 s, all surfaces of the hands)…,” followed by two kinds of situations, i.e., “in the daily life situations (e.g., before preparing food; before eating; after defecation)” or “in disease-related situations (e.g., after blowing nose, coughing, or sneezing; before and after caring for the sick)”
Mask Wearing	Duan et al., 2021; Liang et al., 2021	Two items; 4-point Likert scale from (1) never to (4) always; “During the previous week, I have usually worn a face mask properly…” followed by two different situations relevant to older adults, i.e., “when visiting public places,” and “caring for a person with suspected COVID-19 infection”
Social Distancing	Duan et al., 2021; Liang et al., 2021	Two items; 4-point Likert scale from (1) never to (4) always; “(a) stayed out of crowded places or mass gatherings when going outside of my home, and (b) kept space (at least 1 m) between myself and other people who were coughing or sneezing”
*Past preventive behaviors before the COVID-19 pandemic* Items of each past preventive behavior (hand washing, mask wearing, social distancing) were corresponding to those during the COVID-19 pandemic aforementioned.		
**Motivational factors of preventive behaviors**		
Risk Perception	Perloff and Fetzer, 1986; Duan et al., 2017	Two items; VAS ranging from 0 = lowest to 100 = highest; “How likely do you believe it is for you to become infected with COVID-19 if you do not wash hands frequently/wear face mask/keep a secure social distancing?,” and “Compared to an average person of your age and gender, what is your risk of COVID-19 infection from lack of frequent hand washing/face mask wearing/social distancing?”
Health Knowledge	Contzen and Mosler, 2015; Li et al., 2019	At the beginning, clear instructions of the WHO recommendations for each of the three preventive behaviors were provided, e.g., “According to the WHO recommendations, the proper mask use consists of the following aspects, namely, when and how to wear face masks: (1) if you are taking care of a person with suspected 2019-nCoV infection; (2) if you are coughing or sneezing….” One item; 4-point Likert scale from (1) do not know to (4) know all; “Have you known how and in what situations to wash hands/ wear face mask/ keep a secure social distancing in accordance with the WHO recommendations?”
Attitude	O’Boyle et al., 2001; Rosen et al., 2009	Four items; VAS ranging from 0 = lowest to 100 = highest; a common stem on three preventive behaviors “For me to wash hands frequently/wear face mask/keep a secure social distancing during the outbreak of COVID-19 would be…” followed by 4 bipolar sliders: harmful-beneficial, troubling-reassuring, unpleasant-pleasant, and optional-necessary.
Subjective Norm	Ajzen, 2002; Chung et al., 2018	Two items; VAS ranging from 0 = lowest to 100 = highest; “Most people who are important to me (e.g., my family members, friends, doctors) think that I should wash hands frequently/wear face mask/keep a secure social distancing during the outbreak of COVID-19,” and “Most people who are important to me wash hand frequently/wear face mask/keep a secure social distancing during the outbreak of COVID-19”
Intention	Lippke et al., 2009; Duan et al., 2018; Liang et al., 2019	Two items; VAS ranging from 0 = lowest to 100 = highest; Sample items were “Today and in the near future, I intend to properly wash my hands in daily life situations/wear a face mask when I visit public places/stay out of crowded places or mass gatherings.
Motivational Self-efficacy	Zhou et al., 2016; Duan et al., 2018; Liang et al., 2019	Two items; VAS ranging from 0 = lowest to 100 = highest; “I feel certain that I can begin to wash my hands/wear my mask/keep social distance frequently even if it is time-consuming,” “I feel certain that I can begin to wash my hands/wear my mask/keep social distance even if it may cause inconvenience to my life”
**Volitional factors of preventive behaviors**		
Volitional Self-efficacy	Zhou et al., 2016; Duan et al., 2018; Liang et al., 2019	Two items; VAS ranging from 0 = lowest to 100 = highest; “I feel certain that I can maintain washing my hands frequently/wearing face mask/keeping a secure social distancing even if it takes much time for that to be part of my daily routine.” “I feel certain that I can restart to wash my hands frequently/wear face mask/keep a secure social distancing even if I forgot to do it a few times”
Planning	Luszczynska and Schwarzer, 2003; Duan et al., 2018; Liang et al., 2019; Zhang et al., 2020	Two items; VAS ranging from 0 = lowest to 100 = highest; “I have already made a concrete action plan regarding when, where and how to wash hand /wear face mask/keep social distancing” “I have made a coping plan to maintain frequent hand washing/mask wearing/social distancing if I am confronted with some barriers”
Action Control	Sniehotta et al., 2005; Zhang et al., 2020	Three items; VAS ranging from 0 = lowest to 100 = highest; “I consistently monitor how and in what situations I wash my hands/wear my mask/keep social distance,” “I continuously make sure that I wash my hands/wear my mask/keep social distance properly,” and “I really try hard to wash my hands/wear my mask/keep social distance in necessary situations”

VAS, Visual Analogue Scale.

### Statistical Analysis

Data was analyzed using SPSS 25.0. Normality distribution checks were performed before data analysis. Descriptive analyses, including mean and SD for normal distributed variables, mean rank for non-normal distributed variables, were performed to evaluate the level of the three preventive behaviors and demographic attributes. All variables except chronic disease and BMI [weight in kg/(height in meter)^2^] were transformed into normal ones using Box-Cox (Osborne, 2010). Significant differences in preventive behaviors across some demographic groups (gender, living in or outside Hubei Province, having children or not, restriction/quarantine or not, important others infection of COVID-19 or not) and some other demographic groups (age, living status, marital status, education levels, occupational status, economic situation, self-reported health status, infection of COVID-19 or not) were evaluated using a Mann–Whitney *U* test and a Kruskal–Wallis H test, respectively. *T*-tests were applied to determine if there were significant differences in the three preventive behaviors between those who had chronic diseases or not. Pearson correlation was used to test the relationship between preventive behavior and BMI.

Before the regression analysis, the diagnostics of multicollinearity were performed. The criteria of serious multicollinearity problems include high correlation (*r* > 0.85), low tolerance (≤0.01), high variance inflation factor (VIF > 10), low eigenvalue (approaching zero), and large condition index (>30) among predictors (Duan et al., 2018). Finally, two sets of regressions were employed to examine the associations between relevant factors and each preventive behavior. First, three univariate regressions were used to identify the significant contributing demographic, motivational and volitional variables for each behavior, respectively. Afterwards, multiple hierarchical regressions were conducted with four models for each behavior. In Model 1, the significant demographics were set as predictors. In Model 2, past behavior was set as a predictor. In Model 3 and 4, significant motivational factors and volitional factors were included as predictors step-by-step. The effect size (Cohen *f*^2^; Cohen, 1988) was calculated for further identification of the magnitude of the association between predictors and each preventive behavior. Cohen *f*^2^ of 0.02 is a small effect, 0.15 a medium effect, and 0.35 a large effect. For all analysis, the significance level was set as 0.05 (two-tailed).

## Results

### Sample Characteristics

The descriptive characteristics of the sample are presented in Table 2. The data of 928 eligible older adults were included in the analysis. Most participants were females (55.9%), were aged between 60 and 69 years (70.2%) and were married (85.8%). Most older adults lived in Hubei Province (71.9%) and lived with spouse/partner and/or children at home (91.1%). Only a small percentage of participants were illiterate or semi-illiterate (11.0%), most participants were pensioners/retired (87.7%), and more than half of the sample indicated an average level of household income (55.3%). 98.1% of participants had not been infected with COVID-19, but 6.8% of them reported that their family members, friends, or neighbors had been infected. More than half of the participants (64.1%) were not quarantined. In terms of health status, most participants perceived themselves as satisfactory or excellent (92.1%), and 46.9% of them had chronic disease.

**Table 2:** Descriptive characteristics of the study sample (*n* = 928).

Title		*N* (%)
Age (years), mean (SD): 67.24 (6.43)		
60–69		651 (70.2%)
70–79		223 (24.0%)
80 and above		54 (5.8%)
Gender		
Male		409 (44.1%)
Female		519 (55.9%)
Living place		
In Hubei Province		667 (71.9%)
Outside Hubei Province		261 (28.1%)
Marital status		
Single		7 (0.8%)
In a committed relationship		5 (0.5%)
Married		796 (85.8%)
Divorced		26 (2.8%)
Widowed		94 (10.1%)
Children		
Yes		913 (98.4%)
No		15 (1.6%)
Living status		
Living alone at home		77 (8.3%)
Living alone at nursing home		1 (0.1%)
Living with spouse/partner at home		467 (50.3%)
Living with spouse/partner at the nursing home		4 (0.4%)
Living with spouse/partner and children together		261 (28.1%)
Living with children		118 (12.7%)
Educational level		
Primary school or below		102 (11.0%)
Middle or high school		407 (43.9%)
College or above		419 (45.2%)
Occupational status		
Full time employment		27 (2.9%)
Part-time employment		13 (1.4%)
Pensioner/retired		814 (87.7%)
Unemployed		74 (8.0%)
Economic situation		
Below average		202 (21.8%)
Average		513 (55.3%)
Above average		213 (23.0%)
Restriction/quarantine		
Yes		333 (35.9%)
No		595 (64.1%)
Infection of COVID-19		
Definitely not		910 (98.1%)
I do not know		13 (1.4%)
Definitely yes, the test was positive		5 (0.5%)
Important others infection		
Yes		63 (6.8%)
No		865 (93.2%)
Health status		
Bad		73 (7.9%)
Satisfactory		354 (38.1%)
Excellent		501 (54.0%)
Chronic disease		
Yes		435 (46.9%)
No		493 (53.1%)
BMI, mean (SD): 23.15 (2.78)		
**Preventive behaviors, mean (SD)**		
	**Past preventive behaviors before the COVID-19 pandemic**	**Preventive behaviors during the COVID-19 pandemic**
Hand washing	3.01 (0.74)	3.47 (0.59)
Mask wearing	3.22 (0.85)	3.76 (0.42)
Social distancing	3.18 (0.80)	3.64 (0.48)

### Demographic Characteristics of Hand Washing Behavior

M-W *U* test revealed that hand washing behavior scores of females was significantly higher than that of males (*U* = 90,779, *z* = −4.022, *p* < 0.001) and those who were living in Hubei Province had significantly higher hand washing behavior scores than those outside Hubei (*U* = 78,453, *z* = −2.484, *p* = 0.013; see Table 3). K–W H test revealed that hand washing behavior was significantly different for living situations (*p* = 0.006), educational level (*p* < 0.001), occupational status (*p* < 0.001) and self-rated health status (*p* = 0.009). *Post-hoc* tests showed that the hand washing behavior score of those who were living with spouse/partner at home was significantly higher than that those who were living with spouse/partner and children together (adjusted *p* = 0.012). Scores of those who received college or above education were significantly higher than those who had middle or high school education, as well as primary school education or below, respectively (adjusted *p* = 0.000–0.019). The scores of those who were unemployed were significantly lower than those who were in full-time employment (adjusted *p* = 0.003) and those who were pensioner/retired (adjusted *p* < 0.001). Scores of those who reported bad health status was significantly lower than those who reported as excellent (adjusted *p* = 0.003; see Table 3).

**Table 3:** Demographic characteristics and differences on three preventive behaviors (*n* = 928).

	Hand washing behavior				Mask wearing behavior				Social distancing behavior			
	Mean rank		Adjusted *p*		Mean rank		Adjusted *p*		Mean rank		Adjusted *p*	
Age												
60–69 years old	473.83		0.199		472.96		0.040^*^		471.16		0.221	
70–79 years old	438.81				455.92				456.73			
80 years old and above	458.13				397.91				416.24			
Gender												
Male	426.95		0.000^***^		438.89		0.001^**^		446.37		0.038^*^	
Female	494.09				484.68				478.79			
Living place												
In Hubei Province	477.38		0.013^*^		472.66		0.066		480.30		0.001^**^	
Outside Hubei Province	431.58				443.64				424.11			
Marital status												
Single	375.00		0.870		400.14		0.424		433.36		0.720	
In a committed relationship	517.70				500.10				448.10			
Married	465.54				468.04				468.45			
Divorced	474.96				393.52				469.92			
Widowed	456.63				457.07				432.75			
Children												
Yes	465.27		0.466		465.76		0.168		415.40		0.416	
No	417.40				388.00				465.31			
Living status												
Living alone at home	433.47		0.006^**^		455.88		0.102		427.38		0.183	
Living alone at nursing home	5.50				604.50				654.50			
Living with spouse/partner at home	492.30				480.20				483.43			
Living with spouse/partner at the nursing home	391.38				246.88				396.50			
Living with spouse/partner and children together	426.96				445.78				449.18			
Living with children	464.14				455.60				448.41			
Educational level												
Primary school or below	361.30		0.000^***^		368.94		0.000^***^		370.47		0.000^***^	
Middle or high school	437.42				464.15				461.90			
College or above	515.93				488.11				489.91			
Occupational status												
Full time employment	479.43		0.000^***^		443.39		0.000^***^		414.76		0.000^***^	
Part-time employment	371.81				414.15				380.85			
Pensioner/retired	482.14				476.15				478.23			
Unemployed	281.31				352.93				346.28			
Economic situation												
Below average	199.03		0.116		424.21		0.006^**^		469.29		0.071	
Average	513.70				470.16				422.88			
Above average	217.51				489.06				468.32			
Restriction/quarantine												
Yes	461.50		0.787		464.19		0.974		463.54		0.926	
No	466.18				464.67				465.04			
Infection of COVID-19												
Definitely not	465.37		0.527		465.87		0.108		467.26		0.002^**^	
I do not know	389.08				344.27				238.19			
Definitely yes, the test was positive	501.70				527.20				551.30			
Important others infection												
Yes	462.69		0.953		544.67		0.002^**^		482.04		0.541	
No	464.63				458.66				463.22			
Health status												
Bad	404.45		0.009^**^		454.31		0.146		458.00		0.291	
Satisfactory	446.56				411.51				449.86			
Excellent	485.93				463.00				451.33			
	*M*	**SD**	*p*		*M*	**SD**	*p*		*M*	**SD**	*p*	
Chronic disease												
Yes	3.44	0.62	0.147		3.76	0.42	0.916		3.65	0.50	0.695	
No	3.49	0.55			3.75	0.41			3.64	0.46		
	*M*	**SD**	*r*	*p*	*M*	**SD**	*r*	*p*	*M*	**SD**	*r*	*p*
BMI	23.15	2.78	−0.01	0.755	23.15	2.78	0.031	0.342	23.15	2.78	0.004	0.895

*M*, Mean; SD, standard deviation.

* *p* < 0.05;

** *p* < 0.01;

*** *p* < 0.001.

### Demographic Characteristics of Mask Wearing Behavior

M-W *U* test revealed that mask wearing behavior scores of females was significantly higher than that of males (*U* = 95,660, *z* = −3.197, *p* = 0.001). K–W H test revealed that mask wearing behavior was significantly different for age (*p* = 0.040), educational level (*p* < 0.001), occupational status (*p <* 0.001), and economic situations (*p* = 0.006). Mask wearing behavior scores of those who were aged between 60 and 69 years was significantly higher than that of those who 80 years and above (adjusted *p* = 0.043). The scores of those who had primary school education or below were significantly lower than those who had middle or high school education (adjusted *p* = 0.004) as well as college education or above (adjusted *p* = 0.000). The scores of those who were unemployed was significantly lower than those who were pensioner/retired (adjusted *p* < 0.001). The scores of those who reported that their family economic situation was below average were significantly lower than those who reported being average (adjusted *p* = 0.032) and above average (adjusted *p* = 0.007; see Table 3).

### Demographic Characteristics of Social Distancing Behavior

M-W *U* test revealed that social distancing behavior scores of females was significantly higher than those of males (*U* = 98,719, *z* = −2.078, *p* = 0.038) and those who were living in Hubei Province had significantly higher social distancing behavior scores than those outside Hubei (*U* = 76,503, z = −3.262, *p* = 0.001; see Table 3). Social distancing behavior was significantly different for educational level (*p* < 0.001), professional status (*p* < 0.001) and infection with COVID-19 (*p* = 0.048). Social distancing behavior scores of those who had primary school education or below were significantly lower than those with middle or high school education (adjusted *p* = 0.001) as well as college education or above (adjusted *p* = 0.000). The scores of those who were unemployed was significantly lower than those who were pensioner/retired (adjusted *p* < 0.001). Scores of those who did not know whether they were infected with COVID-19 or not were significantly lower than those who reported definitely not infected (adjusted *p* = 0.002) and those who with a positive test result (adjusted *p* = 0.035; see Table 3).

### Regression Analysis With Three Preventive Behaviors of Older Adults

The multicollinearity diagnostics indicated that there were no serious multicollinearity problems among predictors for the three preventive behaviors. For hand washing behavior, *r* = 0.10–0.55, tolerance = 0.36–0.96, VIF = 1.05–2.73, eigenvalue = 0.01–6.12, and condition index = 1.00–21.76. For mask wearing behavior, *r* = 0.09–0.36, tolerance = 0.15–0.99, VIF = 1.01–6.64, eigenvalue = 0.02–4.42, and condition index = 1.00–14.63. For social distancing behavior, *r* = 0.11–0.36, tolerance = 0.17–0.98, VIF = 1.03–6.01, eigenvalue = 0.02–4.59, and condition index = 1.00–17.69.

### Demographic and Psychological Correlates of Hand Washing Behavior

For hand washing behavior, three univariate regressions identified six out of 15 significant contributing demographic variables [*R*^2^ = 0.111, *F*(5, 927) = 7.593, *p* < 0.001] including gender (*β* = 0.372, *p* < 0.001), living place (*β* = 0.189, *p* = 0.009), living status (*β* = 0.075, *p* = 0.021), educational level (*β* = 0.305, *p* < 0.001), occupational status (*β* = 0.131, *p* = 0.033), and health status (*β* = 0.157, *p* = 0.004), three out of six significant motivational factors [*R*^2^ = 0.209, *F*(5, 927) = 40.531, *p* < 0.001] including risk perception (*β* = 0.089, *p* = 0.006), health knowledge (*β* = 0.131, *p* < 0.001), and intention (*β* = 0.229, *p* < 0.001), and one out of three significant volitional factors [*R*^2^ = 0.202, *F*(5, 927) = 77.751, *p* < 0.001] including planning (*β* = 0.306, *p* < 0.001).

For multiple hierarchical linear regression, significant demographic variables revealed by univariate analyses were first entered into Model 1 (see Table 4). All demographic variables significantly predicted hand washing behavior, *R*^2^ = 0.106, *F*(5, 927) = 18.293, *p* < 0.001. Past hand washing behavior was entered into Model 2 and significantly contributed to this model, *R*^2^ change = 0.239, *F*(6, 927) = 69.302, *p* < 0.001 (see Table 4). The significant motivational factors revealed by univariate analyses were entered into Model 3. Only health knowledge and intention significantly contributed to the model, *R*^2^ change = 0.046, *F*(9, 927) = 58.997, *p* < 0.001. Planning, as the significant volitional factor revealed in univariate analyses was entered into Model 4 and significantly contributed to the model, *R*^2^ change = 0.004, *F*(10, 927) = 54.372, *p* < 0.001. The full model (Model 4) eventually accounted for 39.5% of the variance in hand washing behavior. In addition, the effect size (*f*^2^) of association for each model indicated that Model 1 *f*^2^ = 0.011, Model 2 *f*^2^ = 0.135, Model 3 *f*^2^ = 0.180 and Model 4 *f*^2^ = 0.185, suggesting the largest medium effect of the association (*f*^2^ > 0.15) was in the full model (Model 4; see Table 4).

**Table 4:** Hierarchical regression analysis with hand washing behavior (*N* = 928).

Variables	Model 1		Model 2		Model 3		Model 4	
	*B* (95% CI)	*β*	*B* (95% CI)	*β*	*B* (95% CI)	*β*	*B* (95% CI)	*β*
Gender	0.364 [0.237, 0.491]	0.181^***^	0.173 [0.063, 0.284]	0.086^**^	0.168 [0.061, 0.275]	0.083^**^	0.163 [0.056, 0.270]	0.081^**^
Living place	0.192 [0.055, 0.328]	0.086^**^	0.172 [0.055, 0.289]	0.077^**^	0.110 [−0.005, 0.225]	0.049	0.102 [−0.013, 0.217]	0.046
Living status	0.075 [0.012, 0.138]	0.074^*^	0.057 [0.003, 0.111]	0.056^*^	0.059 [0.007, 0.111]	0.058^*^	0.053 [0.001, 0.105]	0.053^*^
Educational level	0.297 [0.201, 0.393]	0.198^***^	0.154 [0.070, 0.238]	0.103^***^	0.102 [0.019, 0.184]	0.068^*^	0.096 [0.014, 0.178]	0.064^*^
Occupational status	0.137 [0.020, 0.254]	0.074^*^	0.144 [0.044, 0.244]	0.078^**^	0.065 [−0.033, 0.164]	0.035	0.066 [−0.032, 0.165]	0.036
Health status	0.162 [0.064, 0.259]	0.103^**^	0.078 [−0.006, 0.162]	0.050	0.058 [−0.023, 0.139]	0.037	0.057 [−0.024, 0.138]	0.036
Past behavior			0.508 [0.454, 0.563]	0.508^***^	0.427 [0.370, 0.483]	0.427^***^	0.415 [0.358, 0.472]	0.415^***^
Risk perception					0.031 [−0.025, 0.087]	0.031	0.019 [−0.038, 0.076]	0.019
Health knowledge					0.079 [0.015, 0.143]	0.066^*^	0.072 [0.008, 0.136]	0.061^*^
Intention					0.203 [0.144, 0.263]	0.203^***^	0.140 [0.060, 0.220]	0.140^**^
Planning							0.097 [0.015, 0.180]	0.097^*^
*R* ^2^	0.106		0.345		0.391		0.395	
∆*R*^2^			0.239		0.046		0.004	

* *p* < 0.05;

** *p* < 0.01;

*** *p* < 0.001.

### Demographic and Psychological Correlates of Mask Wearing Behavior

For mask wearing behavior, three univariate regressions identified four out of 15 significant demographic variables [*R*^2^ = 0.058, *F*(5, 927) = 3.738, *p* < 0.001] including gender (*β* = 0.223, *p* = 0.001), age (*β* = −0.071, *p* = 0.038), educational level (*β* = 0.154, *p* = 0.003), and important others infection (*β* = −0.318, *p* = 0.015), three out of six significant motivational factors [*R*^2^ = 0.111, *F*(5, 927) = 19.160, *p* < 0.001] including health knowledge (*β* = 0.085, *p* = 0.008), motivational self-efficacy (*β* = 0.136, *p* = 0.013), and intention (*β* = 0.130, *p* = 0.031), and two out of three significant volitional factors [*R*^2^ = 0.135, *F*(5, 927) = 48.005, *p* < 0.001] including planning (*β* = 0.192, *p* = 0.005), and action control (*β* = 0.193, *p* = 0.007).

For multiple hierarchical linear regression, significant demographic variables revealed by univariate analyses were first entered into Model 1 (see Table 5). Only gender, education level and important others infection significantly predicted mask wearing behavior, *R*^2^ = 0.043, *F*(4, 927) = 10.397, *p* < 0.001. Past behavior was entered into Model 2 and significantly contributed to this model, *R*^2^ change = 0.063, *F*(5, 927) = 22.004, *p* < 0.001. The significant motivational factors revealed in univariate analyses were entered into Model 3. All these factors significantly contributed to the model, *R*^2^ change = 0.074, *F*(8, 927) = 25.393, *p* < 0.001. The significant volitional factors revealed by univariate analyses were entered into Model 4 and all these factors significantly contributed to the Model, *R*^2^ change = 0.021, *F*(10, 927) = 23.197, *p* < 0.001. The full model (Model 4) eventually accounted for 20.2% of the variance in mask wearing behavior. In addition, the effect size (*f*^2^) of association for each model indicated that Model 1 *f*^2^ = 0.002, Model 2 *f*^2^ = 0.012, Model 3 *f*^2^ = 0.034 and Model 4 *f*^2^ = 0.043, suggesting the largest small effect of the association (*f*^2^ > 0.02) was in the full model (Model 4; see Table 5).

**Table 5:** Hierarchical regression analysis with mask wearing behavior (*N* = 928).

Variables	Model 1		Model 2		Model 3		Model 4	
	*B* (95% CI)	*β*	*B* (95% CI)	*β*	*B* (95% CI)	*β*	*B* (95% CI)	*β*
Gender	0.234 [0.105, 0.364]	0.116^***^	0.199 [0.073, 0.324]	0.099^**^	0.166 [0.045, 0.286]	0.082^**^	0.154 [0.034, 0.273]	0.076^*^
Age	−0.062 [−0.126, 0.002]	−0.062	−0.016 [−0.079, 0.047]	−0.016	−0.011 [−0.071, 0.050]	−0.011	−0.008 [−0.068, 0.052]	−0.008
Educational level	0.207 [0.109, 0.304]	0.138^***^	0.191 [0.096, 0.285]	0.127^***^	0.111 [0.019, 0.203]	0.074^*^	0.094 [0.003, 0.186]	0.063^*^
Important others infection	−0.332 [−0.584, −0.080]	−0.084^**^	−0.361 [−0.604, −0.117]	−0.091^**^	−0.297 [−0.531, −0.063]	−0.075^*^	−0.296 [−0.527, −0.065]	−0.074^*^
Past behavior			0.258 [0.195, 0.320]	0.258^***^	0.235 [0.175, 0.295]	0.235^***^	0.213 [0.152, 0.273]	0.213^***^
Health knowledge					0.088 [0.011, 0.166]	0.070^*^	0.077 [0.000, 0.153]	0.061^*^
Motivational self-efficacy					0.152 [0.058, 0.245]	0.152^**^	0.049 [−0.053, 0.150]	0.049
Intention					0.118 [0.025, 0.212]	0.118^*^	−0.082 [−0.204, 0.041]	−0.082
Planning							0.139 [0.007, 0.271]	0.139^*^
Action control							0.199 [0.050, 0.348]	0.199^**^
*R* ^2^	0.043		0.107		0.181		0.202	
∆*R*^2^			0.063		0.074		0.021	
Effect size (*f*^2^)	0.002		0.012		0.034		0.043	

* *p* < 0.05;

** *p* < 0.01;

*** *p* < 0.001.

### Demographic and Psychological Correlates of Social Distancing Behavior

For social distancing behavior, three univariate regressions identified three out of 15 significant contributing demographic variables [*R*^2^ = 0.055, *F*(5, 927) = 3.569, *p* < 0.001] including living place (*β* = 0.105, *p* = 0.002), educational level (*β* = 0.103, *p* = 0.003), and occupational status (*β* = 0.099, *p* = 0.003), three out of six significant motivational factors [*R*^2^ = 0.105, *F*(5, 927) = 17.946, *p* < 0.001] including health knowledge (*β* = −0.122, *p* < 0.001), motivational self-efficacy (*β* = 0.151, *p* = 0.016), and intention (*β* = 0.183, *p* = 0.003), and two out of three significant volitional factors [*R*^2^ = 0.109, *F*(5, 927) = 37.564, *p* < 0.001] including planning (*β* = 0.198, *p* = 0.003), and action control (*β* = 0.148, *p* = 0.040).

For multiple hierarchical linear regression, significant demographic variables revealed by univariate analyses were first entered into Model 1 (see Table 6). All demographic variables significantly predicted social distancing behavior, *R*^2^ = 0.039, *F*(3, 927) = 12.410, *p* < 0.001. Past behavior was entered into Model 2 and significantly contributed to this model, *R*^2^ change = 0.115, *F*(4, 927) = 42.020, *p* < 0.001. The significant motivational factors revealed by univariate analyses were entered into Model 3. Only health knowledge and motivational self-efficacy significantly contributed to the model, *R*^2^ change = 0.042, *F*(7, 927) = 32.063, *p* < 0.001. The significant volitional factors revealed by univariate analyses were entered into Model 4 and no factors significantly contributed to the model, *R*^2^ change = 0.008, *F*(9, 927) = 26.201, *p* < 0.001. The full model (Model 4) eventually accounted for 20.4% of the variance in social distancing behavior. In addition, the effect size (*f*^2^) of association for each model indicated that Model 1 *f*^2^ = 0.002, Model 2 *f*^2^ = 0.024, Model 3 *f*^2^ = 0.040 and Model 4 *f*^2^ = 0.043, suggesting the largest small effect of the association (*f*^2^ > 0.02) was in the full model (Model 4; see Table 6).

**Table 6:** Hierarchical regression analysis with social distancing behavior (*N* = 928).

Variables	Model 1		Model 2		Model 3		Model 4	
	*B* (95% CI)	*β*	*B* (95% CI)	*β*	*B* (95% CI)	*β*	*B* (95% CI)	*β*
Living place	0.213 [0.072, 0.354]	0.096^**^	0.218 [0.086, 0.350]	0.098^**^	0.172 [0.042, 0.302]	0.077^**^	0.167 [0.038, 0.297]	0.075^*^
Educational level	0.175 [0.079, 0.271]	0.117^***^	0.107 [0.016, 0.198]	0.071^*^	0.077 [−0.012, 0.167]	0.052	0.065 [−0.025, 0.154]	0.043
Occupational status	0.190 [0.072, 0.309]	0.103^**^	0.169 [0.058, 0.281]	0.091^**^	0.097 [−0.014, 0.208]	0.052	0.084 [−0.027, 0.195]	0.045
Past behavior			0.343 [0.283, 0.403]	0.343^***^	0.293 [0.232, 0.354]	0.293^***^	0.278 [0.217, 0.339]	0.278^***^
Health knowledge					−0.100 [−0.171, −0.030]	−0.084^**^	−0.106 [−0.176, −0.036]	−0.088^**^
Motivational self-efficacy					0.134 [0.031, 0.237]	0.134^*^	0.047 [−0.070, 0.164]	0.047
Intention					0.078 [−0.026, 0.183]	0.078	−0.031 [−0.157, 0.094]	−0.031
Planning							0.098 [−0.039, 0.234]	0.098
Action control							0.124 [−0.018, 0.266]	0.124
*R* ^2^	0.039		0.154		0.196		0.204	
∆*R*^2^			0.115		0.042		0.008	
Effect size (*f*^2^)	0.002		0.024		0.040		0.043	

* *p* < 0.05;

** *p* < 0.01;

*** *p* < 0.001.

## Discussion

The present study revealed that all three individual preventive behaviors dramatically increased among older adults compared to them before the outbreak of COVID-19, suggesting that public health awareness was raised in general during the COVID-19 pandemic. Moreover, results identified the significant contributing role of gender (except social distancing), education level (except social distancing), living status (hand washing only), important others infection (mask wearing only), living place (social distancing only), health knowledge, intention (hand washing only), planning (except social distancing) and action control (mask wearing only) for all three preventive behaviors.

### Demographic Characteristics of Preventive Behaviors

The finding indicated that women took preventive behaviors more frequently than men, suggesting females may be more sensitive and anxious about their health status and more compliant with guidance compared to men (Choi et al., 2020). Compared to older people with lower or no education, highly educated old people are more likely to take preventive behaviors, which is consistent with previous studies (Zhong et al., 2020). Older people who were pensioner/retired are more likely to take preventive behaviors than those who were unemployed, highlighting the stable income or a certain level of economic status facilitates preventive behaviors among older adults (Duan et al., 2021). The findings above revealed that when authorities motivate older adults to enact COVID-19 preventive behaviors, they need to especially focus on elderly men and older adults who are at a lower economic status (e.g., without pension) and with lower education levels. From a social policy aspect, local government can provide relief funding and epidemic prevention appliances (e.g., face masks, disinfection alcohol, and hand sanitizer) for older adults who have economic disadvantages to facilitate their preventive behaviors. In addition, community administrators can organize workshops for older adults who are at lower education levels to increase their health literacy about preventive behaviors (Duan et al., 2021).

In addition, people who live only with spouse/partner at home are more likely to keep their hands hygienic than those who live with both spouse/partner and children. This might be because older couples alone at home are more independently aware of the importance of their hygiene due to lack of support from their adult children during the COVID-19 pandemic (Fu et al., 2020). People whose important others (e.g., family members or friends) were diagnosed with COVID-19 are more likely to wear masks as they need to visit the hospital more frequently, and wearing a mask is the most effective way to reduce the risk of infection (Chhetri et al., 2020). Compared to those outside Hubei Province, residents in Hubei Province are more likely to maintain hand hygiene and to keep social distancing due to the severity of the COVID-19 pandemic in Hubei.

### Past Preventive Behaviors and Psychological Factors of Preventive Behaviors

Findings from the current study showed that past behaviors significantly predicted all three preventive behaviors. Old people who take preventive behaviors prior to the COVID-19 pandemic were more likely to maintain these behaviors during the COVID-19 pandemic, which is consistent with another study on hand washing of Chinese residents, showing that people with healthy habits before outbreak has higher consciousness of self-protection (Zhong et al., 2020). Moreover, the majority of older adults reported that they took all three preventive behaviors during the online survey period with 91.1% of people hand washing frequently, 99% for mask wearing and 98% for secure social distancing. This implies that the older adult sample highly adhere to preventive behaviors even 1–2 months after the end of Wuhan lockdown on 7 April 2020.

When focusing on psychological factors of preventive behaviors, findings showed that health knowledge significantly predicted all three preventive behaviors. Health knowledge played a vital role in the prevention and control of the epidemic. This is consistent with previous study results that health knowledge could increase preventive behaviors (Duan et al., 2017; Li et al., 2019; Zhang et al., 2020; Liang et al., 2021). Health behavior quality will change with health knowledge (Dingman et al., 2020). Handwashing with soap has been ranked the most vital health knowledge for disease control by breaking the chain of transmission among adults (Suen et al., 2019). In our study, almost all older adults reported that they obtained the information *via* TV/mass media (e.g., radio, website), half of them received information from family members/friends/any other acquaintances, one out of five received the information from a healthcare institution (e.g., at the clinic, at the pharmacy). This provides an approach for future preventive behavior intervention among older adults in China. For example, the neighborhood committees could organize workshops and activities through social media to publicize health knowledge and information on preventive behaviors.

In terms of the psychological factors from TPB and HAPA, the importance of factors was different among respective preventive behaviors. Regarding hand washing behavior, intention from TPB and HAPA as well as planning from HAPA were significant psychological predictors. The findings imply the importance of both motivational and volitional factors on hand hygiene enactment of older adults and are in line with previous evidence among diverse populations (Lee and You, 2020; Zhang et al., 2020; Pacholik-Żuromska, 2021). Future interventions might focus on (1) motivating older adults to form sufficient intention of hand washing through building positive attitudes, enhancing self-efficacy, subjective norm, and risk perception (Gaube et al., 2020). (2) Guiding older adults on how to implement hand washing, such as when, where, and how to keep hand hygiene (Zhang et al., 2020).

In relation to mask wearing behavior, planning and action control from HAPA were significant psychological predictors. The results highlight the importance of volitional factors on mask wearing enactment and maintenance in older adults and are consistent with other studies (Zhang et al., 2018), thus future interventions need to educate older adults how to use self-regulation strategies in complying with mask wearing. Such as making plans about when and where to wear masks, keeping constant awareness on mask wearing and monitoring themselves in wearing masks.

Regarding social distancing behavior, no significant psychological predictors from TPB and HAPA were found. The potential reason might be that the enactment of preventive behaviors is influenced by both internal (e.g., intention, planning, action control) and external sources (e.g., cue-to-action, policy and social environment; Fitzsimons and Bargh, 2004; Schwarzer, 2008). For social distancing which must be performed compulsorily in public areas in China during the survey period, the external sources (i.e., government policy) might outweigh the internal sources (e.g., intention, planning, action control), thereby suppress the prediction of internal sources for the mask wearing execution. Nevertheless, this assumption has not been systematically examined in this study and deserves further research.

### Strength and Limitations

Firstly, this study contributes to the knowledge about the demographic features of Chinese older adults’ preventive behaviors during the COVID-19 pandemic. It is helpful for respective national authorities to learn about older peoples’ implementation toward preventive behavior advice. Secondly, the psychological factors of preventive behaviors were adopted in this study based on well-established theories (TPB and HAPA) and previous evidence, which consists of both motivational and volitional phases. Thirdly, identifying the critical psychological factors of preventive behaviors (e.g., health knowledge, intention, planning and action control) is of importance for future psychological practice. That is, authorities may facilitate older people to perform and maintain preventive behaviors in daily life by enhancing those vital psychological factors of behaviors. It is essential to take individual preventive behaviors for long-lasting COVID-19 prevention as the resurgence of infection has occurred several times worldwide and the national-wide vaccination in China is far from prevalent. Fourthly, using electronic technology and social media to deliver online surveys and to reach large sample sizes (*n* = 928) is the strength of this study. Specifically, it was quite challenging to recruit older adults, with lower education levels, who are unfamiliar with online questionnaires. In addition, more than half of older adults live in Hubei Province, which was the most severely affected region during the COVID-19 pandemic in China and was under strict travel restriction during the survey period. This increased the difficulty of data collection, but from another point of view, it added to the value for this study.

Limitations of the current study should also be acknowledged. Firstly, although the sample size was sufficiently large, it should be recognized that there was some unbalance in the numbers of participants in or outside Hubei Province. Secondly, older adults who were at low socioeconomic status or have visual or reading disabilities may have no access to mobile phones or computers to participate in this online survey. Thirdly, snowball sampling was applied to recruit older adults from Hubei province and outside Hubei province in China, which may weaken the representativeness of samples and findings. Fourthly, self-reported measures of behaviors might be affected by participants’ ability to recall their actions. Finally, the cross-sectional design neglected the effect of time and cannot rigorously reveal the causal relationship between preventive behaviors and their psychological factors. Future studies should enlarge sample size, employ randomized sampling approaches, and administrate both online and offline surveys to enhance the generalization and consider applying longitudinal designs to examine causality relationships.

## Conclusion

The current study revealed that older adult sample in China who are female, with higher education levels, living with only their spouse/partner at home, and live in Hubei Province are more likely to take preventive behaviors during the COVID-19 pandemic. In addition, gender, living status, past behavior, health knowledge, intention, and planning significantly predicted hand washing behavior. Gender, education level, important others infection, past behavior, health knowledge, planning and action control significantly predicted mask-wearing behavior. Living place, past behavior and health knowledge significantly predicted social distancing behavior. Overall, this study demonstrated the demographic features of three preventive behaviors among older adults in China. Identifications of the critical demographic and psychological factors associated with three preventive behaviors can facilitate older adults to perform and adhere to these behaviors in daily life in order to prevent COVID-19 infections.

## Data Availability Statement

The original contributions presented in the study are included in the article/supplementary material, further inquiries can be directed to the corresponding author.

## Ethics Statement

The studies involving human participants were reviewed and approved by Hong Kong Baptist University Research Ethics Committee (REC/19-20/0490). The patients/participants provided their written informed consent to participate in this study.

## Author Contributions

YD, CH, and WL conceived and designed the study. YD, WL, CH, BS, JH, and ZL contributed to the preparation of study materials. YD, ZL, CH, WL, JH, YW, and BS collected the data. CH, JH, and WL screened and analyzed the data. CH and YD drafted the manuscript. YD, JB, and WL revised and polished the manuscript. YD, CH, and WL had full access to all study data and take responsibility for the integrity of the data and the accuracy of the data analysis. All authors reviewed and approved the final version of the manuscript.

## Funding

This research was supported by Start-up Grant and Strategic Development Fund (SDF) of Hong Kong Baptist University. The funding organization had no role in the study design, study implementation, data collection, data analysis, manuscript preparation, or publication decision. The work is the responsibility of the authors.

## Conflict of Interest

The authors declare that the research was conducted in the absence of any commercial or financial relationships that could be construed as a potential conflict of interest.

## Publisher’s Note

All claims expressed in this article are solely those of the authors and do not necessarily represent those of their affiliated organizations, or those of the publisher, the editors and the reviewers. Any product that may be evaluated in this article, or claim that may be made by its manufacturer, is not guaranteed or endorsed by the publisher.
